# Significance of urinary albumin excretion in patients with cast nephropathy

**DOI:** 10.5414/CN109630

**Published:** 2019-06-24

**Authors:** Ryo Nakagawa, Aya Imafuku, Eiko Hasegawa, Hiroki Mizuno, Masahiro Kawada, Akinari Sekine, Rikako Hiramatsu, Keiichi Sumida, Masayuki Yamanouchi, Noriko Hayami, Tatsuya Suwabe, Junichi Hoshino, Naoki Sawa, Kenmei Takaichi, Atsushi Wake, Kenichi Ohashi, Takeshi Fujii, Yoshifumi Ubara

**Affiliations:** 1 Nephrology Center,; 2 Department of Hematology,; 3 Department of Pathology,; 4 Okinaka Memorial Institute for Medical Research, Toranomon Hospital, Tokyo, and; 5 Department of Pathology, Yokohama City University, Graduate School of Medicine, Yokohama, Japan

**Keywords:** urinary albumin excretion, renal biopsy, myeloma cast nephropathy, amyloidosis, multiple myeloma, lymphoplasmacytic lymphoma/Waldenström macroglobulinemia

## Abstract

Background: This study was performed to determine whether the urinary albumin excretion rate (%UAE) could distinguish myeloma cast nephropathy (MCN) without glomerular amyloid deposition from MCN with glomerular amyloid deposition. Materials and methods: We retrospectively reviewed clinicopathological data on 16 patients with MCN diagnosed by renal biopsy at Toranomon Hospital from 2004 to 2014. Results: A total of 10 patients had pure MCN without glomerular amyloid deposition (group 1), and 6 patients had MCN with glomerular amyloid deposition (group 2). In all 10 patients from group 1, the underlying disease was multiple myeloma (MM), while 4 patients had MM, and 2 patients had lymphoplasmacytic lymphoma/Waldenström macroglobulinemia (LPL/WM) in group 2. Total protein did not show a significant difference between the two groups, but serum albumin was significantly higher in group 1 than group 2 (p = 0.0101). Serum-adjusted calcium did not show a significant difference between the groups, while serum creatinine (Cre) was significantly higher in group 1 than group 2 (p = 0.0343). Although urinary protein excretion did not differ significantly between the groups, the %UAE was significantly lower in group 1 than group 2 (p = 0.00198). In group 2, 3 of the 4 patients with MM died within 15 months of diagnosis, but the 2 patients with LPL/WM are alive after 32 months. In group 1, only 1 patient died (of unknown causes) within 15 months after diagnosis. Conclusion: In patients with MCN, %UAE may be a useful marker for the detection of coexistence of glomerular lesions, such as amyloidosis, which are associated with a poor outcome.

## Introduction 

Renal biopsy is considered to be important for the diagnosis of renal involvement in patients with multiple myeloma (MM) because of the therapeutic and prognostic implications. Korbet and Schwartz [1] reviewed renal biopsy data on renal disease in MM patients, revealing that 40 – 63% had myeloma cast nephropathy (MCN), 19 – 26% had light chain deposition disease (LCDD), 7 – 30% had amyloidosis, and less than 1% had cryoglobulinemic renal disease [1, 2]. According to these studies, renal pathology in patients with MM shows a single pattern. However, two patterns of renal disease occurring in the same patient have been reported by other authors. Lin et al. [3] reported a combination of MCN and LCDD in 11 patients, while Lorenz et al. [4] reported a 40-year-old woman whose renal biopsy revealed three concurrent pathologies, including MCN, amyloidosis, and LCDD. While MCN is the most common kidney disease associated with MM, it has also been reported to occur in patients with lymphoplasmacytic lymphoma/Waldenström macroglobulinemia (LPL/WM) [5, 6]. 

Because renal biopsy is invasive and carries a risk of bleeding, a noninvasive diagnostic method for renal disease associated with plasma cell dyscrasias is required. Albuminuria is a useful marker for glomerular involvement. Accordingly, this study was performed to determine whether the urinary albumin excretion rate (%UAE) could be used to distinguish pure MCN from MCN associated with glomerular lesions such as deposition of amyloid. 

## Materials and methods 

### Patient selection 

Renal biopsy was performed in 2,284 patients at Toranomon Hospital from 2004 to 2014. Patients were excluded if they had monoclonal immunoglobulin deposition disease (MIDD) including LCDD, light and heavy chain deposition disease (LHCDD) and heavy chain deposition disease (HCDD), or if they had primary light chain (AL) and heavy chain (AH) amyloidosis, which did not fit the diagnostic criteria for MM [7] or LPL/WM [8]. 16 patients with a diagnosis of MCN were analyzed retrospectively. They were divided into a group that had pure MCN without glomerular amyloid deposition (group 1) and a group with CN plus glomerular amyloid deposition (group 2). This study was approved by the Institutional Review Board (IRB) of Toranomon Hospital, and the patients gave full written informed consent. 

### Histological evaluation 

Renal biopsy specimens were processed for light microscopy (LM), immunofluorescence microscopy (IF), and electron microscopy (EM) according to the previously reported methods [9]. LM specimens were stained with hematoxylin and eosin (H & E), periodic acid Schiff (PAS), periodic acid methenamine silver (PAM), Masson trichrome (MT), and Congo red stain. IF was performed to detect immunoglobulin (Ig)G, IgA, IgM, κ light chain, and λ light chain as well as for complement components (C)1q, C3, and C4. Amyloid was diagnosed by detection of amorphous lesions showing Congo red positivity on LM that contained randomly-arranged fibrils 7 – 12 nm in diameter on EM. 

Tubular injury ratio (tubulointerstitial fibrosis and atrophy/the renal cortex) was classified into mild degree (< 25%), moderate degree (25 – 50%), and severe degree (> 50%). Glomerular sclerotic ratio (global sclerotic glomeruli/total glomeruli × 100, %) was examined. 

### Laboratory tests 

Blood and urine samples were collected from all patients before renal biopsy. Laboratory tests were performed to measure the hemoglobin (g/dL), white blood cell count (×10^4^/μL), platelet count (×10^4^/μl), total protein (TP) (g/dL), serum albumin (g/dL), serum creatinine (Cre) (mg/dL), serum adjusted calcium (a-Ca= serum calcium + 0.8 × (4 – serum) albumin) (mg/dL), serum β2 microglobulin (β2MG), IgG (mg/dL), IgA (mg/dL), IgM (mg/dL), cryoglobulin, antinuclear antibody, 50% hemolytic complement (CH50) (normal range: 32 – 47 U/mL), and estimated glomerular filtration rate (eGFR) (mL/min/1.73m^2^). By 24-hour urine collection, the total protein (g/day), urinary albumin excretion rate (%UAE= percentage of total urinary protein excreted as albumin), urinary free light chain excretion (FLC), and β2MG excretion (μg/day) were measured. In addition, the urine sediment was examined to determine the number of erythrocytes per high-power field (HPF) and other parameters. Serum monoclonal protein and urinary Bence-Jones protein were analyzed by protein electrophoresis and/or immunofixation electrophoresis. 

### Diagnosis of MM and LPL/WM 

Diagnosis of MM was performed according to the criteria of the International Myeloma Working Group [7], and the Durie-Salmon (DS) staging system was employed to assess and classify MM [10]. LPS/WM was diagnosed on the basis of monoclonal IgM gammopathy and bone marrow involvement by LPL (characterized by low-grade B-cell malignancy composed of small lymphocytes and plasmacytoid lymphocytes) [8]. The M protein type was determined by serum and/or urine protein electrophoresis. 

### Diagnosis of myeloma cast nephropathy (MCN) 

MCN was diagnosed in MM or LPL/WM patients with renal dysfunction if renal biopsy revealed casts in the renal tubules that were pale or negative on PAS staining, eosinophilic on H & E staining, and polychromatic (red and blue) on MT staining. These tubular casts were restricted to either κ or λ light chain and were surrounded by inflammatory cells, including plasma cells and lymphoid cells [11]. 

### Statistical analysis 

Data were compiled and analyzed by using EZR (Saitama Medical Center, Jichi Medical University, Saitama, Japan), a graphical user interface for R (The R Foundation for Statistical Computing, Vienna, Austria) [12]. Results were summarized as percentages, as the mean ± standard deviation [SD], or as the median and interquartile range (IQR), unless indicated otherwise. 

## Results 

### Case 1 (pure MCN) 

**Figure 1.**
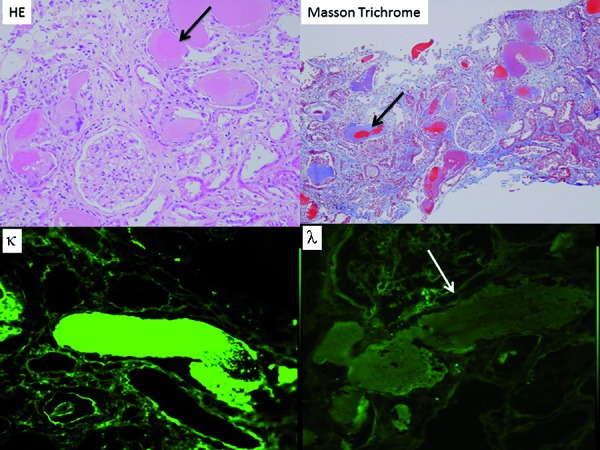
Renal biopsy of case 1 (patient 7 in Table 1). Light microscopy shows a tubular cast surrounded by inflammatory cells. The cast is eosinophilic on H & E staining (arrow), polychromatic (red and blue) on Masson trichrome staining (arrow), positive for κ light chain (arrow), and negative for λ light chain (arrow).

A 56-year-old Japanese woman was admitted to our hospital for evaluation of renal dysfunction with a serum Cre of 5.3 mg/dL and hypercalcemia (a-Ca: 13.5 mg/dL) (Table 1, patient 7). Urinary protein excretion was 18.3 g daily, and urinary Bence Jones Protein (BJP)-κ was positive, while %UAE was only 0.9%. Bone marrow aspiration showed 58.0% κ-positive plasma cells, and MM was diagnosed (DS stage III A). A renal biopsy specimen was obtained, and LM revealed global sclerosis in 1 out of 19 glomeruli. While the glomeruli showed no significant abnormalities, tubular casts (eosinophilic on H & E staining, negative for PAS stain, polychromatic on MT staining, and positive for κ light chain) surrounded by inflammatory cells were detected (Figure 1). Congo red stain was negative throughout the biopsy specimen, so pure MCN was diagnosed. She received three courses of vincristine-doxorubicin (adriamycin)-dexamethasone (VAD) therapy followed by high-dose melphalan (HDM) + autologous peripheral blood stem cell transplantation (SCT). Hematologic remission was not complete, so chemotherapy was continued, but the patient died of subdural hemorrhage 11 years and 2 months after the diagnosis. 

### Case 2 (CN plus AL amyloidosis) 

**Figure 2.**
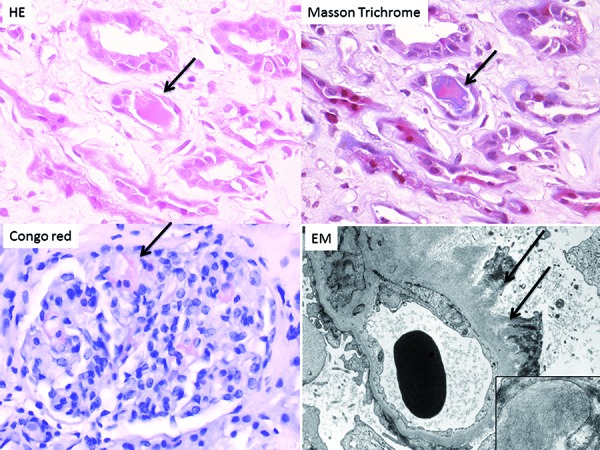
Renal biopsy of case 2 (patient 13 in Table 1). Light microscopy displays tubular casts surrounded by inflammatory cells. Casts are eosinophilic on H & E staining (arrow) and polychromatic (red and blue) on Masson trichrome staining (arrow). There is a Congo red-positive amorphous glomerular lesion (arrow). Periodic acid methenamine silver staining shows subepithelial spicule formation (arrow). Inset: EM shows randomly arranged fibrils measuring 7 – 12 nm in diameter.

A 68-year-old Japanese man was admitted for evaluation of intestinal ileus (Table 1, patient 13). Serum Cre was 1.1 mg/dL, and he had hypercalcemia (a-Ca: 11.8 mg/dL). Urinary protein excretion was 6.7 g daily, with both serum IgA-λ M protein and urinary BJP-λ being positive. %UAE was 59.3%. Bone marrow aspiration showed 31.6% λ-positive plasma cells, and MM was diagnosed (DS stage III-A). Examination of a renal biopsy specimen revealed no global sclerosis in 3 glomeruli on LM. The glomeruli contained Congo red-positive amorphous lesions, and subepithelial spicule formation was seen by PAM staining. In addition, EM revealed randomly-arranged fibrils of 7 – 12 nm in diameter. Tubular casts surrounded by inflammatory cells were seen, which were negative for PAS, eosinophilic on H & E staining, polychromatic on MT staining, and positive for λ light chain (Figure 2). Coexistence of MCN and light chain amyloidosis was diagnosed. Treatment with VAD + prednisolone was started, followed by 2 courses of thalidomide. However, remission was not achieved, and the patient died of pneumonia after 6 months. 

### Clinicopathological features of CN 

Clinicopathological features of the MCN patients are listed in Table 1. The 16 patients consisted of 7 men and 9 women, with a mean (± SD) age of 60.8 ± 9.2 years. 

Group 1 (pure MCN): BJP MM was the most common type in 6 patients (κ in 3 and λ in 3), followed by IgA-κ MM in 3 patients and IgD-λ MM in 1 patient. The D-S stage was I in 1 patient, II in 2 patients, and III in 7 patients. 

Group 2 (MCN plus amyloid deposition): BJP-λ MM was diagnosed in 2 patients, while 2 patients had IgA-λ MM, and 2 patients had IgM-κ LPL/WM. Amyloid deposits were detected in the glomeruli of all 6 patients. While amyloid deposit was confirmed in the heart in 2 patients, in the stomach in 1 patient, in the large intestine in 3 patients, and in the nervous system in 1 patient. 

In group 1, the chief complaints at diagnosis included bone pain (due to bone involvement by MM) in 6 patients, fatigue in 2 patients, and no symptoms (elevation of serum Cre) in 2 patients. In group 2, the chief symptoms were anasarca (due to severe albuminuria) in 5 patients and fatigue in 1 patient. 

Histologically, group 1 included tubular injury associated with MCN of more than moderate degree in a total of 10 patients, and group 2 included tubular injury of mild degree in 4 patients out of 6 patients. Glomerular sclerotic ratio was not significantly different between the two groups. 

### Laboratory data 

Laboratory data are compared between group 1 and group 2 in Table 2. TP did not show a significant difference between the two groups, but serum albumin was significantly higher in group 1 than group 2 (p = 0.0101) (median (IQR) serum albumin was 3.68 (3.2 – 4.3) vs. 2.45 (1.85 – 3.3)). Serum a-Ca did not differ significantly between both groups. Serum Cre was significantly higher in group 1 compared with group 2 (p = 0.0343) (median (IQR) serum Cre was 4.29 (1.58 – 4.85) vs. 0.96 (0.78 – 4.28)). Although urinary protein did not show a significant difference between the two groups, %UAE was significantly lower in group 1 than group 2 (p = 0.00198) (median (IQR) %UAE was 3.55 (1.6 – 6.25) vs. 64.6 (35.2 – 74.00)). Urinary FLC (g/day) was significantly higher (p = 0.0146) in group 1 compared with group 2. 

### Treatment 

Treatment is summarized in Table 1. In group 1, 5 patients received SCT after conditioning with VAD regimen and HDM. Thalidomide (Thal) was added in 3 patients, and melphalan prednisolone (MP) was added in 1 patient. MP only was given to 1 patient, while 1 patient received MP + VAD (4 cycles) + bortezomib (Bor) (3 cycles), 2 patients were given dexamethasone (DEX) + Bor, and 1 patient was treated with VAD (1 cycle) and MP (4 cycles). 

In group 2, 1 patient received MP alone (6 cycles), 1 patient was treated with rituximab (RTX) + DEX (1 cycle) + cyclophosphamide + bortezomib + dexamethasone (CyBorD) (1 cycle), 1 patient was given VAD + Thal, 1 patient had VAD alone (2 cycles), 1 patient received RTX + DEX + CyBorD, and 1 patient was given SCT + RTX. 

### Outcome 

In group 1 (pure MCN), only 1 patient died (of unknown causes) less than 15 months after diagnosis. In group 2 (CN with amyloid deposition), 3 patients with MM died after less than 15 months, and 1 MM patient is alive after 28 months. The 2 patients with LPL/MW are alive after 32 months. 

## Discussion 

In patients with MCN, tubular obstruction is caused by light chain casts, which form when monoclonal light chain production leads to high urinary excretion of free light chains that undergo coprecipitation with Tamm-Horsfall protein in the distal tubules [13]. 

Albuminuria is a useful indicator of glomerular disease. Leung et al. [14] compared %UAE among pure MCN (n = 43) with AL-amyloidosis (n = 177), LCDD (n = 28), and acute tubular necrosis (n = 12). In the patients with pure MCN, %UAE was significantly lower than in the patients with glomerular lesions (AL amyloidosis or LCDD), but they did not perform a comparison between pure MCN and MCN with amyloid deposition. 

Nasr et al. [15] evaluated 190 patients with MM who underwent renal biopsy at the Mayo Clinic. The most common paraprotein-associated lesions were MCN (33%), monoclonal immunoglobulin deposition disease (22%), and amyloidosis (21%), while the main non-paraprotein-associated lesions were acute tubular necrosis (9%), hypertensive arteriosclerosis (6%), and diabetic nephropathy (5%). %UAE was highest in amyloidosis and lowest in MCN. Median survival from the diagnosis of MM was 44, 58, and 62 months in patients with MCN, amyloidosis, and monoclonal immunoglobulin deposition disease, respectively (p = 0.4) . 

Although detection of MCN on renal biopsy is usually related to MM, CN has also been found in patients with LPL/WM. Perez et al. [5] reported a 76-year-old woman with LPL/WM who had monoclonal gammopathy (IgM-κ isotype) and cervical lymphadenopathy (biopsy showed monotonous proliferation of small lymphocytes with lymphoplasmacytoid differentiation). In this patient, renal biopsy showed evidence of light chain CN and the diagnosis was CN associated with LPL/WM. In addition, Gnemmi et al. [6] reported CN plus LCDD in 2 patients with LPL/WM. 

In conclusion, we assessed the clinicopathological features and laboratory data of 16 patients with CN. %UAE may be a useful marker for the coexistence of glomerular amyloid deposition in patients with CN, which is important because coexisting amyloidosis seems to be related to a poor prognosis. In group 1 (pure MCN without amyloidosis), MM-related bone involvement may be the chief complication, and renal complications (tubulointerstitial MCN) may be detected at a later stage (eGFR: 19.5 mL/min/1.73 m^2^, CKD 4). In group 2 (MCN with amyloidosis), tubular damage was mild, and glomerular damage due to amyloid deposition could contribute to nephrotic range albuminuria and the occurrence of anasarca at an earlier stage of kidney disease (eGFR: 53.2 mL/min/1.73 m^2^, CKD 3A). Tubular injury associated with MCN might contribute to renal deterioration on group 1. 

### Limitation 

There are some limitations in this study. This was a small study. We could not discuss whether LPL/WM plus amyloidosis patients have a better prognosis than MM patients with amyloidosis, although LPL/WM patients with CN plus amyloidosis were shown to have better outcomes than MM patients with MCN plus amyloidosis because treatments used in this study might not be the most effective treatments for MM or amyloidosis and could result in inappropriate outcomes from suboptimal treatments. 

%UAE of patient 10 in group 1 showed 14.9%, while %UAE of patient 11 in group 2 showed 13%. The difference of these 2 patients could not be differentiated. But the other 9 patients in group 1 showed %UAE less than 7.7%, and the other 5 patients in group 2 showed %UAE of more than 42.5%. This will indicate that patients with MCN with %UAE of more than 42.5% may have glomerular disease including amyloidosis, and MCN with %UAE less than 7.7% may become pure MCN. On the other hand, it is possible that %UAE may be the result of the severe renal impairment because there was a difference of renal function between group 1 and group 2. However, there is a report supporting our opinion. Leung et al. [14] reported that %UAE of MCN shows 7%, while %UAE of AL amyloid shows 70%. 

## Funding 

This study was funded by the Okinaka Memorial Institute for Medical Research. 

## Conflict of interest 

The authors declare that they have no financial or other conflicts of interest in relation to this research and its publication. 

**Table 1.** Clinicopathological features of CN (both pure MCN and MCN+ amyloid deposition).

Group		Patient No	Age	Sex	Complaints	DS	sCre (mg/dL)	UP (g/day)	%UAE	IgG	IgA	IgM	Serum M protein	Urine M protein	Amyloid	Sclerotic glomeruli (%)	Tubular injury ration (%)	Treatment	Follow up	Outcome
Group 1 (pure CN)	MM	1	72	F	No symptom	DS II, ISS III	1.5	5	5.4	602	73.1	23.7	ND	BJP-λ	ND	21/46 (45)	25 – 50%	MP^(3)^	22	Another hospital
		2	65	F	Bone pain	DSIIIB	1.5	5.3	5.8	632	19.5	11.1	ND	BJP-κ	ND	2/25 (8)	25 – 50%	MP^(1)^, VAD^(4)^, Bor^(3)^	144	Doing well
		3	76	F	Bone pain	DSIIIB	1.9	3.2	1	559	41.8	9.1	ND	BJP-λ	ND	10/28 (35.7)	25 – 50%	DEX + Bor	36	Died of sepsis
		4	62	F	Bone pain	DSIIIA	2.2	10.6	3.1	607	99.3	10.8		BJP-κ	ND	2/16 (12.5)	> 50%	VAD^(1)^, MP^(4)^	1	Another hospital
		5	47	M	No symptom	DSIIIB	2.7	1	7.6	548	644.4	15.7	IgA-λ	IgA-λ	ND	10/35 (28.6)	> 50%	VAD3, SCT^(2)^, Thal	142	Doing well
		6	57	M	Bone pain	DSIIIB	4.6	6.5	4	231	11.7	8	IgD-λ	BJP-λ	ND	2/42 (4.7)	> 50%	VAD3, SCT^(2)^, Thal	29	Died of relapse of MM
		7	56	F	General fatigue	DSIIIB	5.6	18.2	0.9	472	28.7	8.5	ND	BJP-κ	ND	1/19 (5.2)	> 50%	VAD3, SCT^(2)^, Thal	125	Died of subdural hemorrhage
		8	58	M	Bone pain	DSIIIB	12.1	8.55	1.8	188	463.3	5.4	IgA-λ	BJP-λ	ND	0/10 (0.0)	> 50%		17	Died of relapse of MM
		9	53	M	Bone pain	DSIB	12.6	2.3	3.1	389	36.6	10.8	ND	BJP-λ	ND	2/8 (25)	> 50%	VAD, SCT^(2)^, MP	96	Another hospital
		10	47	M	General fatigue	DSIIB	13.1	6	14.7	371	477.3	8.8	IgA-λ	BJP-λ	ND	10/35 (28.6)	> 50%	VAD3, SCT	28	Died of unknown cause
Group 2 (CN plus amyloid)	MM	11	71	F	General fatigue	DSIIA	0.7	2.7	13	1.397	45.9	46.5	ND	BJP-λ	K, H, S	3/18 (16.7)	< 25%	MP^(6)^	15	Died of amyloid
		12	68	F	Anasarca		0.8	3.86	69.9	531	548.8	69.6	IgA-λ	BJP-λ	K, S, I	6/28 (21.7)	< 25%	RTX + DEX, CyBorD	32	Doing well
		13	68	M	Anasarca	DSIIIA	1.1	6.7	59.3	510	1.860	12.1	IgA-λ	BJP-λ	K, I	0/3 (0.0)	< 25%	VAD, Thal	6	Died of pneumonia
		14	58	F	Anasarca	DSIII	13.2	6.6	42.6	297	10.4	9.4	ND	BJP-λ	K, H, I, N	0/10 (0.0)	> 50%	VAD^(2)^	10 M	Died of heart disease
	LPL/WM	15	77	F	Anasarca		0.82	1.85	75.9	814	123	709.9	IgM-κ	BJP-λ	K	4/41 (9.8)	< 25%	RTX + DEX, CyBorD	43	Doing well
		16	63	M	Anasarca		1.31	4.64	73.3	40	98.4	791.5	IgM-κ	BJP-λ	K	11/34 (37.9)	25 – 50%	SCT, RTX	32	Doing well

MM = multiple myeloma; LPL/WM = lymphoplasmacytic lymphoma/Waldenström macroglobulinemia; MCN = myeloma cast nephropathy; K = kidney involvement; H = heart; S = stomach; I = large intestine; N = nerves; SCT = autologous peripheral blood stem cell transplantation; Dex = dexamethasone; Bor = bortezomib; Thal = thalidomide; CyBorD = cyclophosphamide + bortezomib + dexamethasone; ND = not detected. MP = melphalant prednisolone; VAD = vinchristine-doxurubicin(adriamycin)-dexamethasone; RTX = rituximab. ^(1)^^(2)^^(3)^^(4)^^(6)^ = Number of cycles.

**Table 2.** Laboratory data of patients with pure MCN and MCN + amyloid deposition.

Group	1	2	
Variables	Pure CN (n = 10)	CN plus amyloid (n = 6)	Significant difference
Sex (M/F)	M5/F5	M2/F4	
Age	57.6 (51.5 – 68.7)	68 (61.75 – 72.5)	0.082
κ/λ	κ2/λ4	κ3/λ7	
TP (g/dL)	6.35 (5.9 – 7.23)	6.1 (4.85 – 7.53)	0.584
Serum Alb (g/dL)	3.68 (3.2 – 4.3)	2.45 (1.85 – 3.3)	0.0101
Serum β 2MG (mg/L)	7.15 (4.55–22.5)	5.35 (2.73 – 11.68)	0.232
BUN (mg/dL)	29 (26 – 41)	18.5 (16 – 36.5)	0.0925
Serum Cre (mg/dL)	4.29 (1.58 – 4.85)	0.96 (0.78 – 4.28)	0.0343
eGFR (mL/min/1.73m^2^)	19.5 (10.4 – 27.2)	53.2 (33.7 – 59.2)	0.0312
UA (mg/dL)	7.35 (6.8 – 7.98)	7.15 (5.18 – 9.33)	0.704
Serum a-Ca (mg/dL)	9.55 (9.3 – 11.3)	10.45 (10.08 – 10.98)	0.158
Hb (g/dL)	8.35 (6.88 – 11.1)	11.3 (9.0 – 12.45)	0.212
Urinary protein (g/day)	5.65 (2.98 – 9.06)	4.25 (2.49 – 6.62)	0.492
%UAE (%)	3.55 (1.6 – 6.25)	64.6 (35.2 – 74.00)	0.00198
Urinary FLC (g/day)	4.55 (2.82 – 8.02)	1.5 (0.72 – 2.48)	0.0146
	Median (IQR)	Median (IQR)
